# Supplementary material for: Ordinary kriging vs inverse distance weighting: spatial interpolation of the sessile community of Madagascar reef, Gulf of Mexico
Source: PeerJ. 2017 Nov 30;5:e4078. doi: 10.7717/peerj.4078 (PMC5712470; doi:10.7717/peerj.4078)
Supplement: Data S2 — Parameters of the best models of inverse distance weighting (IDW) and ordinary kriging (OK) for the interpolation of macroalgae, octocorals, sponges, Millepora alcicornis (millepora) and zoanthids of Madagascar reef, Gulf of Mexico. [file peerj-05-4078-s004.docx]

|  | **Macroalgae** | **Octocorals** | **Sponges** | **Millepora** | **Zoanthids** |
| --- | --- | --- | --- | --- | --- |
| **OK and IDW Searching Window Parameters** | | | | | |
| **Points per Sector** | 5 | 5 | 5 | 5 | 5 |
| **Number of Sectors** | 4 | 4 | 4 | 4 | 4 |
| **Angle** | 9 | 11 | 9 | 13 | 9 |
| **Major axis (m)** | 10 | 10 | 5 | 10 | 15 |
| **Minor Axis (m)** | 150 | 150 | 145 | 150 | 130 |
| **IDW Weighting Exponentials** | | | | | |
|  | 2 | 2 | 2 | 2 | 2 |
| **OK Variography** | | | | | |
| **Number of Lags** | 12 | 12 | 12 | 10 | 10 |
| **Lag size (m)** | 7 | 5 | 4 | 5 | 5 |
| **Nugget** | 0.010 | 0.006 | 0.0011 | 0.0011 | 0.002 |
| **Model** | Exponential | Spherical | Spherical | Spherical | Exponential |
| **Anisothropy** | Yes | Yes | Yes | Yes | Yes |
| **Major Range** | 60 | 70 | 48 | 49 | 50 |
| **Minor Range** | 20 | 50 | 20 | 14 | 12 |
| **Direction (degrees)** | 365 | 9 | 205 | 19 | 18 |
| **Sill** | 0.058 | 0.030 | 0.001 | 0.001 | 0.002 |

Supplementary Table 1. Parameters of the best models of Inverse Distance Weighting (IDW) and Ordinary Kriging (OK) for the interpolation of Macroalgae, Octocorals, Sponges, *Millepora alcicornis* (Millepora) and Zoanthids of Madagascar reef, Gulf of Mexico.
